# Public Perception of Health Care Before, During, and After COVID-19: Longitudinal Analysis of Online Reviews

**DOI:** 10.2196/96824

**Published:** 2026-08-12

**Authors:** Javier Gazquez-Garcia, Carlos Luis Sánchez-Bocanegra, Carlos Fernandez-Llatas

**Affiliations:** 1 Institute for Information and Communication Technologies (ITACA), SABIEN Group, Universitat Politècnica de València, Camino de Ver, S/N, Valencia, 46022, Spain, 1 963 87 70 00; 2 Distrito Sanitario Almería, Servicio Andaluz de Salud, Ctra. de Ronda, 226, 2º Planta, Almeria, Andalusia, 04009, Spain, 1 950 18 68 19; 3 Faculty of Computer Science, Multimedia and Telecommunications, Universitat Oberta de Catalunya, Barcelona, Spain

**Keywords:** patient experience, COVID-19, online reviews, primary care, hospitals, infodemiology

## Abstract

**Background:**

Online reviews of health care services represent a growing source of unsolicited, citizen-generated data that can complement traditional instruments for monitoring public perception of health systems. However, longitudinal analyses examining how citizens’ perceptions evolved before, during, and after the COVID-19 pandemic remain scarce, and existing studies have rarely differentiated between levels of care.

**Objective:**

This study aimed to examine the longitudinal evolution of public perception of a regional public health system over a 10-year period, with particular attention to differences between primary care and hospital services, and to assess whether the COVID-19 pandemic produced a temporary disruption or a more persistent structural shift in citizens’ evaluation of health care.

**Methods:**

A retrospective longitudinal observational study was conducted using 47,589 online reviews from 812 public health care facilities in Andalusia, Spain, collected from Google Maps between 2016 and 2025. Reviews were classified as positive or negative based on star ratings and validated against manual annotation using Cohen κ. The proportion of negative reviews was analyzed across 3 periods: prepandemic (2016‐2019), pandemic (2020‐2022), and postpandemic (2023‐2025). Structural breaks were identified through change-point detection analysis. Logistic regression models with robust standard errors clustered at the facility level were used to quantify differences in negative sentiment across levels of care and over time.

**Results:**

The proportion of negative reviews increased from 38.7% (2598/6713) in the prepandemic period to 73.7% (13,296/18,046) during the pandemic, remaining elevated at 66.5% (15,181/22,830) in the postpandemic period. Change-point detection identified March 2020 as a major structural break. The pandemic had markedly different effects across levels of care: negative reviews in primary care rose from 34.8% (1330/3819) to 81.9% (10,138/12,371) during the pandemic, remaining at 75.7% (9464/12,505) postpandemic, whereas hospital care showed a more moderate increase from 43.8% (1268/2894) to 55.6% (3158/5675), remaining stable thereafter. Logistic regression models confirmed that the trajectory of negative perception in primary care diverged significantly from hospital care during and after the pandemic, with interaction terms indicating substantially higher odds of negative reviews in primary care during the pandemic (odds ratio 5.28, 95% CI 3.95‐7.07) and postpandemic period (odds ratio 3.66, 95% CI 2.66‐5.03).

**Conclusions:**

The findings indicate that the COVID-19 pandemic was associated with a persistent structural shift in public perception of health care services rather than a temporary fluctuation and that this shift was disproportionately concentrated in primary care. The sustained deterioration in citizens’ perception of primary care observed years after the acute crisis suggests that postpandemic recovery strategies should explicitly address the postcrisis phase and prioritize the relational and communicative dimensions of primary care alongside structural capacity. Large-scale digital trace data offer a scalable and continuous complement to traditional patient satisfaction instruments for monitoring health system legitimacy over time. These findings should be interpreted in light of the self-selection bias inherent to online reviews and the single-region scope of the analysis.

## Introduction

### Online Reviews as a Source of Citizen Perception Data

Over the past decade, the evaluation of health systems has increasingly extended beyond traditional survey instruments toward large-scale digital traces of citizen experience, particularly online reviews of health care organizations and professionals [1]. Platforms such as Google Maps, Yelp, and physician-rating websites have turned citizens from passive respondents into active evaluators whose unsolicited, free-text assessments accumulate continuously over multiple years [2-5]. This makes them particularly well suited for studying temporal changes in public perception of health care services [6].

Throughout this manuscript, the terms experience, perception, and sentiment are used to refer to conceptually distinct but related layers of citizen engagement with health services. Experience refers to the actual encounter between a citizen and the health care system. Perception refers to how that experience is interpreted, evaluated, and remembered by the individual, shaped by expectations, prior interactions, and broader institutional narratives. Sentiment refers to the affective valence expressed in observable discourse, including online reviews and ratings, constituting the empirical layer accessible to large-scale digital trace analysis. The present study operates at the level of sentiment as an empirically observable proxy for perception, while acknowledging that this proxy is mediated by the conditions under which reviews are produced and shared.

The unstructured nature of these textual narratives requires computational methods to be analyzed at scale. Sentiment classification and change-point detection procedures allow researchers to transform free-text content into structured longitudinal indicators of population-level perception, complementing conventional measures of system performance with insights derived directly from citizen discourse [7-12].

### Public Perception and the Legitimacy of Health Systems

Public perception of health care services plays a critical role in shaping trust in health institutions and the legitimacy of publicly funded systems [13]. Trust has been associated with important behavioral outcomes, including the willingness to seek care, adherence to medical advice, and public support for collective financing arrangements that sustain universal health coverage [14]. Conversely, persistent narratives of dissatisfaction or institutional failure may erode confidence in public systems, contribute to the avoidance of services or bypassing of primary care, and influence broader societal debates regarding the organization and financing of health care [5].

Importantly, experiences and expectations of care differ across levels of the health system. Primary care typically functions as the main entry point to publicly funded systems and is characterized by frequent, routine interactions with citizens, longitudinal relationships, and coordination of care [2]. Hospital care, by contrast, is usually experienced through episodic encounters, often in contexts of acute illness or heightened vulnerability [15]. These structural differences generate distinct evaluative frameworks: primary care tends to be judged in terms of accessibility, continuity, and relational quality, whereas hospital services are more often evaluated with regard to perceived technical competence, safety, and management of complex clinical situations [12]. Consequently, different levels of the same health system may produce distinct experiential trajectories and narrative patterns in citizen discourse [16].

### COVID-19 as a Systemic Organizational Shock

The COVID-19 pandemic produced an unprecedented organizational shock to publicly funded health systems, forcing rapid reconfiguration of services, clinical pathways, and resource allocation [17,18]. Across many countries, including Spain, this translated into the near-overnight substitution of face-to-face encounters with telephone or digital consultations in primary care, the postponement of routine non-COVID services, and altered accessibility and continuity of care for large segments of the population [19-21].

Emerging empirical studies suggest that the first waves of the pandemic were associated with a deterioration in patient-reported experience, particularly in access, communication, and waiting times, and that online reviews and patient complaints documented increasingly negative narratives during the acute phase [22,23]. However, most existing analyses rely on cross-sectional designs or limited observation windows surrounding the acute crisis, and longitudinal analyses of citizen discourse spanning several years before and after the pandemic remain scarce [24].

### The Gap in the Literature

Although the literature documents important disruptions in patient experience during the pandemic, several fundamental questions remain unresolved from a longitudinal perspective [24]. It remains unclear whether public perception of health care services has returned to prepandemic levels or has stabilized at a new plateau of negativity. Likewise, little is known about whether deterioration in public perception reflects a temporary adjustment or a more durable shift in how citizens evaluate health services. Furthermore, the extent to which primary and hospital care have followed similar or divergent trajectories of perception after COVID-19 has not been systematically examined.

### Conceptual Framework: Temporary Shock vs Structural Transformation

From a conceptual standpoint, 2 broad interpretative models can be considered when examining postpandemic trajectories in citizen perception of health systems. In a temporary shock model, the pandemic is understood as producing an abrupt but transient disturbance: levels of dissatisfaction increase during the acute crisis but gradually return to baseline once organizational pressures diminish and services recover [25]. In this scenario, the pandemic does not fundamentally alter the structure of citizen discourse about health care; it merely amplifies preexisting concerns temporarily.

In contrast, a structural transformation model conceptualizes the pandemic as a critical juncture that reshapes how citizens interpret and evaluate their encounters with health services. Under this model, the crisis may lead to persistent increases in negative perception, reordering of thematic priorities in patient narratives, and potentially widening differences in how various levels of the health system are evaluated [26]. From this perspective, the pandemic does not simply perturb evaluative patterns but contributes to a more enduring reconfiguration of expectations, problematization, and legitimacy surrounding health care services.

### Study Objective and Research Question

To address this gap, the present study analyzes a longitudinal corpus of 47,589 online reviews of public health care facilities in Andalusia (Spain), spanning from 2016 to 2025, drawn from a larger initial pool of over 66,000 reviews retrieved from Google Maps and filtered for textual content, length, and language consistency. Using validated sentiment classification and change-point detection, the analysis examines whether the COVID-19 pandemic produced a temporary disruption or a more persistent structural shift in public perception of health services, with particular attention to differences between primary care and hospital care.

## Methods

### Study Design

This study was designed as a retrospective longitudinal observational study based on user-generated online reviews. The objective was to analyze the evolution of public perception of the Andalusian public health system using large-scale textual data generated by patients on digital platforms.

The analysis included reviews of public primary care centers and hospitals that belong to the Andalusian Public Health System (Servicio Andaluz de Salud). By covering both levels of care, the study aimed to capture potential differences in public perception across components of the health system. The longitudinal nature of the dataset enabled analysis of temporal changes in perception over several years.

The design and reporting of this study adhered to the STROBE (Strengthening the Reporting of Observational Studies in Epidemiology) statement [27]. A completed STROBE checklist is provided as Checklist 1.

### Ethical Considerations

This study was based exclusively on publicly available, user-generated content posted on Google Maps. No interaction with patients or health care professionals took place, no clinical or otherwise identifiable health data were accessed, and no informed consent was required.

The study protocol (study code AP-0020 2025) was submitted to the Provincial Research Ethics Committee of Almería (Comité de Ética de la Investigación con medicamentos, CEIm Provincial de Almería), an accredited committee constituted in accordance with Andalusian Decree 8/2020 of 30 January (Official Gazette of the Regional Government of Andalusia [BOJA] number 24, 5 February 2020) [28]. On 26 November 2025, the committee issued a formal ruling (reference SICEIA-2025‐002988) determining that the study did not require ethics committee evaluation (“no procede valoración por parte del CEI/CEIm”) on the grounds that it did not involve human participants in the regulatory sense established by Spanish Law 14/2007 of 3 July [29] on Biomedical Research and relied exclusively on publicly available secondary data.

Reviewer names and any other potentially identifying information were excluded from the analytical dataset; only the textual content, star ratings, dates, facility identifiers, and aggregated geographic information were retained. Data processing complied with European General Data Protection Regulation (EU-GDPR) 2016/679 [30] and Spanish Organic Law 3/2018 [31].

### Data Source and Data Collection

The sampling frame included all publicly funded primary care and hospital facilities within the Andalusian Public Health System, identified through the official institutional directory of the Andalusian Health Service. This publicly available registry was exported to a structured dataset containing facility names and identifiers, which served as the basis for the automated identification of the corresponding locations on Google Maps.

Because the objective was to analyze citizens’ perceptions of the public health system as a whole, all facilities listed in the directory were included and no sampling procedures were applied. Consequently, no a priori sample size calculation was performed: the analytical corpus reflects a population-based census of all publicly available digital reviews of the regional public health care system rather than a probabilistic sample.

### Web Scraping Procedure

Data collection followed a multistage automated web scraping pipeline. A custom Python script based on the Selenium framework searched for each facility on Google Maps and retrieved the corresponding location URLs. These URLs were then processed using an automated scraping tool implemented on the Apify platform, which extracted all publicly available reviews associated with each location, including review text, star rating, review date, reviewer name, overall location rating, geographic coordinates, and facility metadata.

The extraction was conducted in December 2025 and retrieved all reviews available on the platform at that time, with the earliest reviews dating back to 2010. To ensure temporal consistency and sufficient data density for longitudinal analysis, the analytical period was restricted to reviews published between 2016 and 2025.

### Text Preprocessing and Data Cleaning

Although the primary analysis relied on star ratings as a proxy for sentiment, textual data were processed to ensure data quality and to enable validation procedures.

Reviews that did not contain textual content were removed. In addition, reviews containing fewer than 5 words were excluded in order to eliminate entries that did not provide meaningful narrative information.

The remaining reviews were subjected to standard text-cleaning procedures, including conversion to lowercase, removal of punctuation, removal of URLs and special characters, and filtering using regular expressions.

Language detection procedures were applied to exclude reviews written in languages other than Spanish, ensuring linguistic consistency within the dataset.

The initial scraping pipeline retrieved approximately 66,000 raw reviews across the registered facilities. After excluding reviews without textual content, reviews with fewer than 5 words, and reviews not written in Spanish, the final analytical corpus consisted of 47,589 textual reviews from 812 publicly funded health care facilities. A summary of the cascade of inclusion and exclusion criteria is provided in Multimedia Appendix 1 (flow diagram). The full text of all retained reviews was preserved in the analytical dataset.

### Sentiment Categorization

For the purpose of analyzing public perception, reviews were categorized according to their star rating, which served as a proxy indicator of sentiment. Reviews with ratings of 4 or 5 stars were classified as positive, whereas reviews with ratings of 1 or 2 stars were classified as negative. Reviews with 3 stars were classified as neutral and were retained in the analytical dataset; in the binary logistic regression models, they were coded as not negative.

To assess the validity of this proxy, a subsample of 300 reviews was drawn from the analytical corpus through simple random sampling, without stratification by year, level of care, or star rating. Each review was manually classified by the first author as positive, negative, or neutral based on the affective valence of the review text, with the label reflecting the dominant tone of the narrative. Agreement between the star-rating–based classification and the manual annotation was quantified using Cohen κ coefficient, yielding κ=0.84, which supports the use of star ratings as a scalable proxy indicator of sentiment in large-scale observational datasets [32].

### Temporal Analysis

To examine temporal changes in public perception, the proportion of negative reviews was calculated for each year of the study period. Temporal trends were visualized using annual time-series plots comparing primary care and hospital services.

The study period was divided into 3 phases: prepandemic (2016‐2019), pandemic (2020‐2022), and postpandemic (2023‐2025), enabling comparisons across different stages of the COVID-19 pandemic.

The 2023‐2025 period is operationalized as postpandemic with reference to the formal end of the World Health Organization Public Health Emergency of International Concern for COVID-19 on 5 May 2023 [33]. Although major nonpharmaceutical interventions in Spain had already been progressively relaxed during 2022, that year was retained within the pandemic period because it represented a transitional phase in which health care services continued to adapt to pandemic-related organizational disruptions and changing patterns of care delivery. We acknowledge that the boundary between the pandemic and postpandemic phases is not sharp and that some endemic dynamics of SARS-CoV-2 transmission persist; the operational definition used here reflects the institutional and organizational transition from emergency response to routine care management.

### Change-Point Detection

To identify potential structural breaks in the temporal trajectory of negative perception, a change-point detection analysis was conducted using the pruned exact linear time algorithm implemented in the ruptures Python library.

The algorithm was applied to the monthly time series of negative review proportions using a radial basis function cost function, allowing the detection of distributional changes over time.

### Statistical Analysis

Logistic regression models were used to quantify differences in negative sentiment across levels of care and over time, with negative sentiment as the dependent variable.

The analysis followed a stepwise approach. First, a baseline model assessed the association between level of care (primary vs hospital) and the probability of a review being negative. Second, a model including year (centered) was introduced to account for temporal trends in sentiment. Third, an interaction model incorporating level of care × year interaction was specified to examine whether temporal trajectories differed between primary and hospital care.

To specifically evaluate the impact of the COVID-19 pandemic, an additional model included pandemic period categories (prepandemic, pandemic, and postpandemic) and interaction terms between the level of care and the pandemic period.

The use of progressively specified nested models, together with the 2 complementary parameterizations of time (centered year as a continuous trend and pandemic period as a categorical exposure), served as a robustness check for the stability of the main associations across alternative analytical strategies.

Results were reported as odds ratios (ORs) with 95% CI. Robust standard errors clustered at the health care facility level were used for inference on coefficients, while likelihood ratio tests were used to compare nested models.

All reviews retained in the analytical corpus had complete data for the variables used in the statistical models (star rating, date, facility identifier, and level of care). Reviews with missing text or with fewer than 5 words had been excluded during preprocessing, and no imputation of missing data was therefore required at the analytical stage.

### Software Environment

All analyses were conducted in Python. Data manipulation and preprocessing used pandas and NumPy; logistic regression modeling and additional statistical procedures used statsmodels and SciPy; change-point detection was performed with the ruptures library; text processing for cleaning and validation relied on NLTK and spaCy; and data visualization was implemented with Matplotlib and Seaborn.

## Results

### Data Collection and Review Retrieval

**Figure 1.**
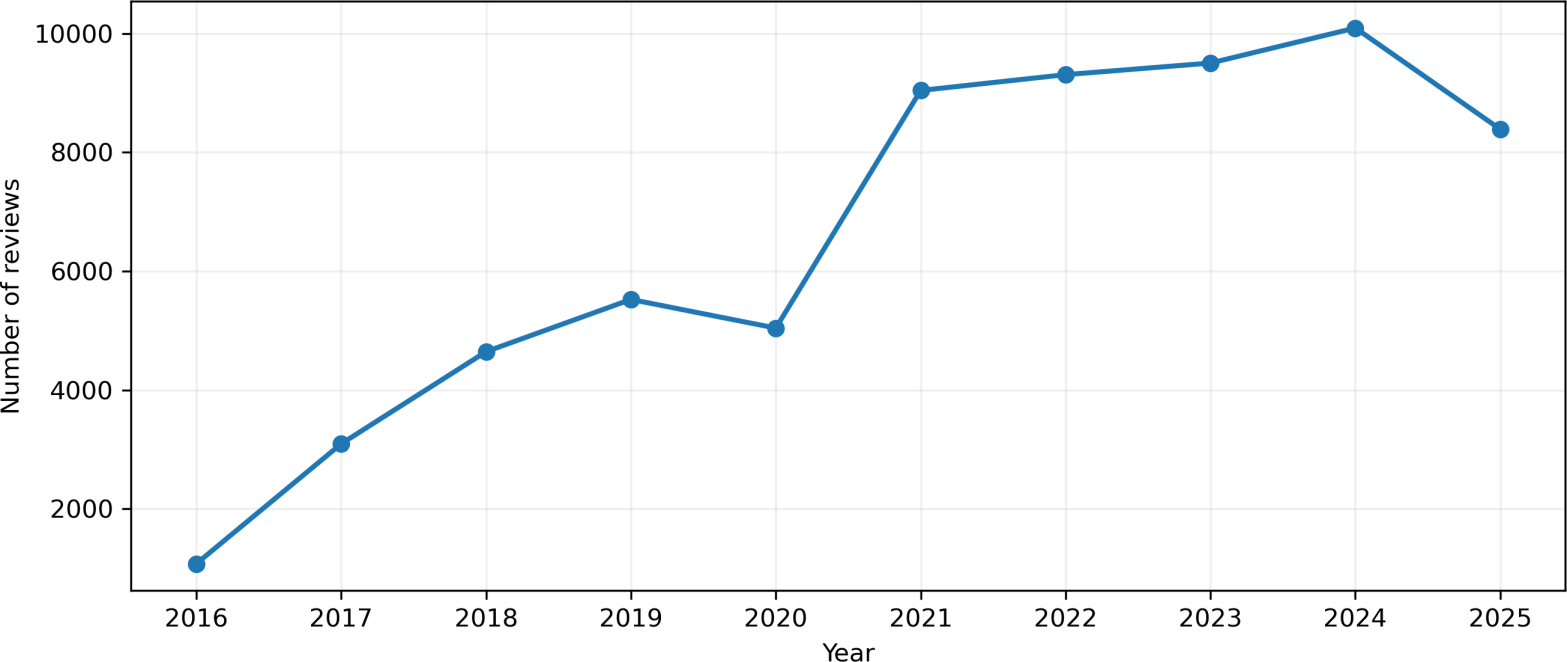
Annual volume of raw user-generated online reviews retrieved from Google Maps for public health care facilities of the Andalusian Public Health System (Servicio Andaluz de Salud), Spain, 2016‐2025.

The data collection pipeline retrieved all available online reviews associated with the identified public health care facilities during the extraction process. Figure 1 shows the annual volume of raw reviews retrieved before the application of text-length and language eligibility criteria, illustrating the progressive increase in the use of online platforms for sharing health care experiences over the study period. After preprocessing and the application of the inclusion criteria, the final analytical corpus consisted of 47,589 textual reviews from 812 publicly funded health care facilities of the Andalusian Public Health System.

Across the analytical corpus of 47,589 reviews, 65.3% (n=31,075) of reviews were classified as negative, 30.4% (n=14,470) as positive, and 4.3% (n=2044) as neutral, according to the sentiment categorization based on star ratings.

### Temporal Evolution of Negative Perception

The proportion of negative reviews varied substantially across the study period. Prior to the COVID-19 pandemic, negative reviews accounted for approximately 38.7% (12,026/31,075) of annual reviews. During the pandemic period, this proportion increased markedly to 73.7% (22,902/31,075). Although the proportion declined slightly in the postpandemic period, it remained elevated at 66.5% (20,664/31,075), well above prepandemic levels.

**Figure 2.**
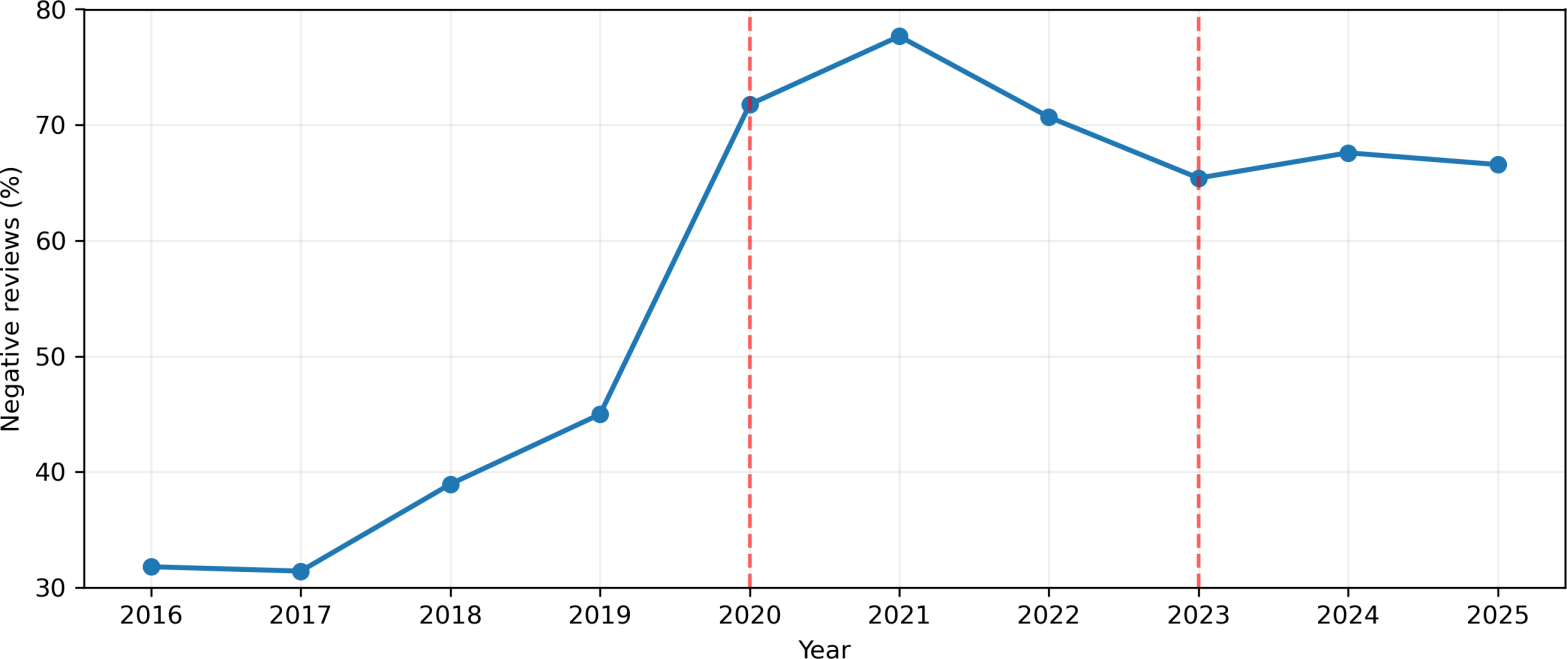
Temporal evolution of the proportion of negative online reviews of public health care facilities in the Andalusian Public Health System, Spain, 2016‐2025 (retrospective longitudinal observational study, 47,589 user-generated reviews from 812 facilities via Google Maps). Reviews were classified as negative when assigned 1 or 2 stars by the reviewer. Vertical dashed lines indicate the boundaries of the COVID-19 pandemic period, with the prepandemic period defined as 2016‐2019, the pandemic period as 2020‐2022, and the postpandemic period as 2023‐2025.

Overall, the proportion of negative reviews did not return to prepandemic levels in the most recent years of the dataset, suggesting a sustained shift in citizens’ perceptions following the pandemic rather than a purely temporary fluctuation (Figure 2).

### Change-Point Detection

**Figure 3.**
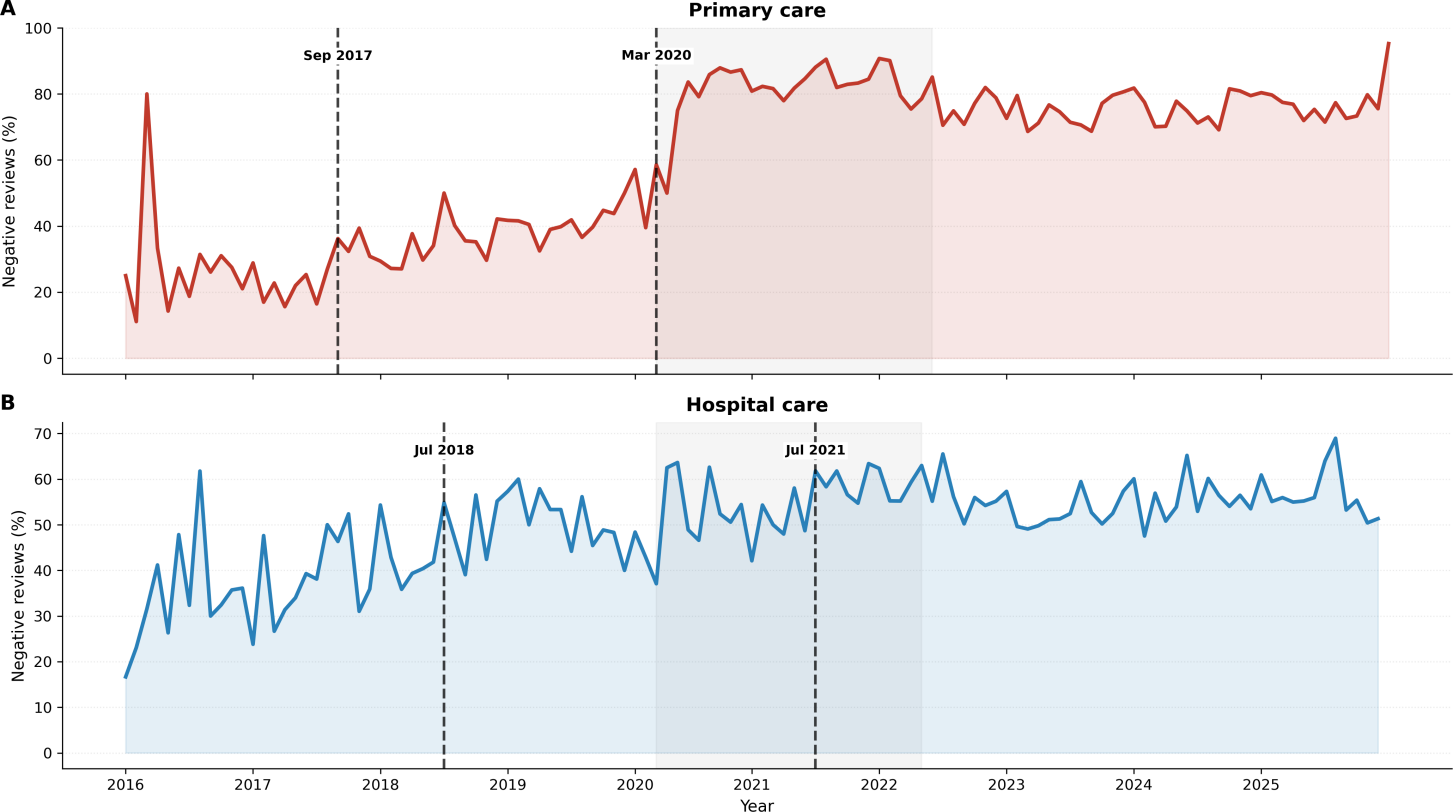
Structural change-point detection in the monthly proportion of negative online reviews of public health care facilities in the Andalusian Public Health System, Spain, 2016‐2025 (retrospective longitudinal observational study, 47,589 user-generated reviews from 812 facilities via Google Maps). Reviews were classified as negative when assigned 1 or 2 stars. Dashed vertical lines indicate structural change points detected using the pruned exact linear time (PELT) algorithm with a radial basis function (RBF) cost function, as implemented in the ruptures Python library. The shaded area corresponds to the COVID-19 pandemic period.

The change-point detection analysis identified 3 structural change points in the trajectory of negative reviews. The most prominent was detected around March 2020, coinciding with the onset of the COVID-19 pandemic and marking a sharp increase in the proportion of negative reviews. Additional change points were identified around September 2017 in primary care and around July 2018 in hospital care, suggesting earlier shifts in the dynamics of negative perception prior to the pandemic (Figure 3).

To quantify the magnitude of these shifts, the proportion of negative reviews was compared across the prepandemic, pandemic, and postpandemic periods (Table 1). In primary care, negative reviews increased markedly from 34.8% (1330/3819) in the prepandemic period to 81.9% (10,138/12,371) during the pandemic, remaining elevated at 75.7% (9464/12,505) in the postpandemic period. In contrast, hospital care showed a more moderate increase, from 43.8% (1268/2894) before the pandemic to 55.6% (3158/5675) during the pandemic, remaining relatively stable thereafter (5717/10,325, 55.4% in the postpandemic period). The pandemic had a substantially stronger impact on the perceived quality of primary care services, with negative perceptions remaining persistently higher even after the acute pandemic phase.

**Table 1.** Proportion of reviews classified as negative across prepandemic, pandemic, and postpandemic periods, stratified by level of care, in public health care facilities in the Andalusian Public Health System (Servicio Andaluz de Salud), Spain^a^.

Period	Primary care, n/N (%)	Hospital care, n/N (%)
Prepandemic (2016‐2019)	1330/3819 (34.8)	1268/2894 (43.8)
Pandemic (2020‐2022)	10,138/12,371 (81.9)	3158/5675 (55.6)
Postpandemic (2023‐2025)	9464/12,505 (75.7)	5717/10,325 (55.4)

a Based on a retrospective longitudinal observational analysis of 47,589 user-generated reviews from 812 facilities retrieved from Google Maps. Reviews were classified as negative when assigned 1 or 2 stars by the reviewer.

### Logistic Regression Models

The proportions reported in the preceding subsections describe the absolute magnitude of negative perception within each level of care and time period, providing a descriptive picture of how citizen-generated sentiment varies across the study period. The logistic regression models presented in this subsection complement these descriptive proportions by quantifying the relative strength of the associations between negative sentiment, level of care, and time, while accounting for the nonindependence of reviews nested within facilities using robust standard errors clustered at the facility level. The ORs reported below should therefore be interpreted as adjusted measures of relative differences, not as estimates of absolute prevalence.

In the baseline model including only the level of care, reviews referring to primary care showed significantly higher odds of being negative compared with hospital reviews (OR 2.33, 95% CI 1.98‐2.73; *P*<.001).

After adjusting for temporal variation using the centered year variable, the association between level of care and negative sentiment remained statistically significant (OR 2.48, 95% CI 2.12‐2.90; *P*<.001). In addition, the year variable showed a positive association with negative sentiment (OR 1.15, 95% CI 1.12‐1.19; *P*<.001), indicating that the probability of negative reviews increased progressively over time.

A third model including an interaction term between level of care and time was estimated to assess whether the difference between primary and hospital care evolved across the study period. The interaction term was statistically significant (OR 1.15, 95% CI 1.09‐1.21; *P*<.001), indicating that the increase in the probability of negative reviews over time was more pronounced in primary care compared with hospital care (Table 2).

**Table 2.** Logistic regression models estimating the probability of a review being classified as negative as a function of level of care (primary care vs hospital services) and time^a^.

Variable	Model 1^b^	Model 2^c^	Model 3^d^
Primary care, OR^e^ (95% CI)	2.33 (1.98‐2.73)	2.48 (2.12‐2.90)	2.53 (2.18‐2.94)
Year (centered), OR (95% CI)	—^f^	1.15 (1.12‐1.19)	1.08 (1.04‐1.11)
Primary care × year, OR (95% CI)	—	—	1.15 (1.09‐1.21)
Pseudo-*R*²	0.030	0.048	0.052

a Based on a retrospective longitudinal observational analysis of 47,589 user-generated online reviews of 812 public health care facilities of the Andalusian Public Health System, Spain, 2016‐2025 (Google Maps). Reviews were classified as negative when assigned 1 or 2 stars by the reviewer; hospital care served as the reference category for level of care.

b Model 1 includes only level of care.

c Model 2 adjusts for temporal trend using centered year variable.

d Model 3 adds an interaction term between level of care and the centered year variable.

e Odds ratios with 95% CI are reported, with robust standard errors clustered at the health care facility level.

f Not applicable.

**Figure 4.**
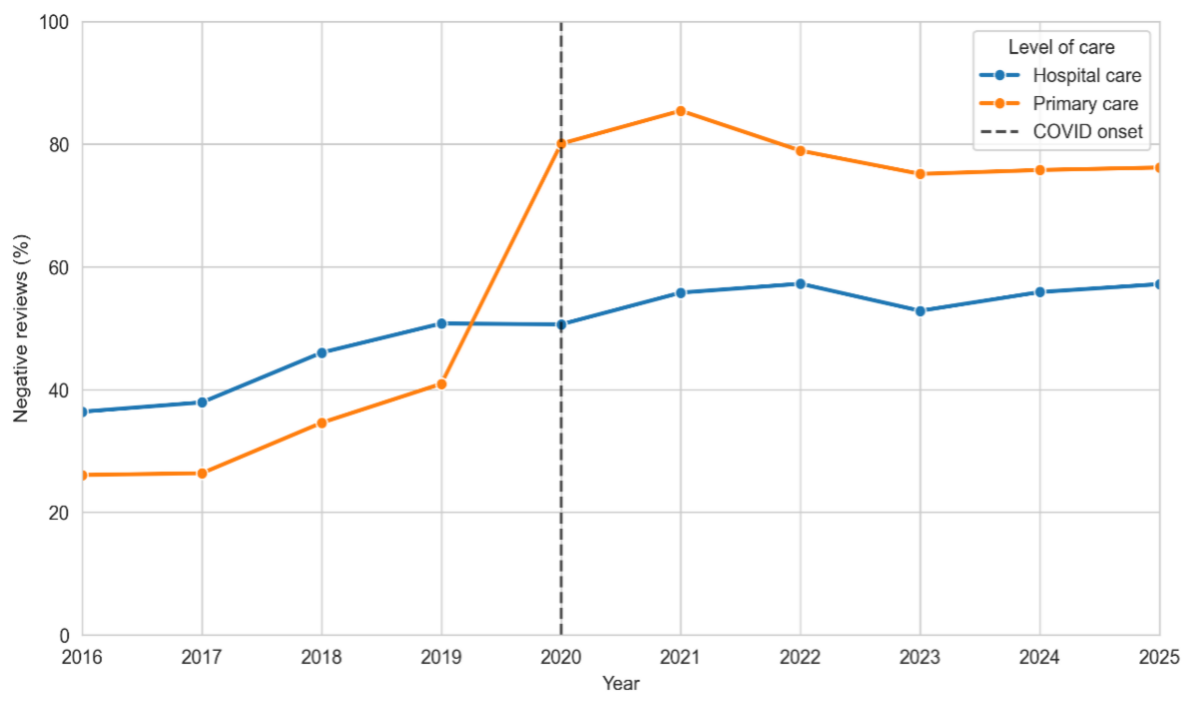
Temporal evolution of the proportion of negative online reviews stratified by level of care (primary care vs hospital services) in the Andalusian Public Health System, Spain, 2016‐2025 (retrospective longitudinal observational study, 47,589 user-generated reviews from 812 facilities via Google Maps). Reviews were classified as negative when assigned 1 or 2 stars by the reviewer. The dashed vertical line indicates the onset of the COVID-19 pandemic in Spain (March 2020), which coincides with the structural break identified by the change-point detection analysis.

A separate set of logistic regression models incorporated the pandemic period as a categorical exposure along with its interaction with level of care. Compared with the prepandemic reference, the odds of negative reviews increased during the pandemic (OR 1.61, 95% CI 1.26‐2.05; *P*<.001) and remained elevated in the postpandemic period (OR 1.59, 95% CI 1.23‐2.06; *P*<.001). While primary care showed lower odds than hospital care in the prepandemic period (OR 0.69, 95% CI 0.51‐0.91; *P*=.009), it experienced a markedly sharper increase during and after the pandemic, with statistically significant interaction terms (Table 3). A likelihood ratio test confirmed that the interaction model improved fit relative to the baseline model (LR=3144.83, *df*=4, *P*<.001), indicating that the association between level of care and negative sentiment varied significantly across pandemic periods. Taken together, these findings indicate that the COVID-19 pandemic had a disproportionate and persistent impact on public perception of primary care services (Figure 4).

**Table 3.** Logistic regression model assessing the association between level of care (primary care vs hospital services), pandemic period (prepandemic 2016‐2019, pandemic 2020‐2022, and postpandemic 2023‐2025), and the probability of a review being classified as negative^a^.

Variable	OR^b^ (95% CI)	*P* value
Primary care	0.69 (0.51‐0.91)	.009
Pandemic period	1.61 (1.26‐2.05)	<.001
Postpandemic period	1.59 (1.23‐2.06)	<.001
Primary care × pandemic	5.28 (3.95‐7.07)	<.001
Primary care × postpandemic	3.66 (2.66‐5.03)	<.001

a Based on a retrospective longitudinal observational analysis of 47,589 user-generated online reviews from 812 public health care facilities in the Andalusian Public Health System, Spain (Google Maps). Hospital care and the prepandemic period served as reference categories. Reviews were classified as negative when assigned 1 or 2 stars by the reviewer.

b Odds ratio with 95% CI and *P* values are reported, with robust standard errors clustered at the health care facility level.

## Discussion

### Principal Findings

This longitudinal analysis of citizen-generated online reviews of the Andalusian public health care system over a 10-year period yielded 3 principal findings, each directly addressing the objectives stated in the *Introduction* section. First, the proportion of negative reviews increased sharply from 38.7% in the prepandemic period to 73.7% during the pandemic and remained elevated at 66.5% in the postpandemic period, with change-point detection identifying March 2020 as a major structural break in the monthly time series. Second, the pandemic affected primary care and hospital services in markedly distinct ways: negative reviews in primary care rose from 34.8% to 81.9% during the pandemic and persisted at 75.7% thereafter, whereas hospital care showed a more moderate and stable shift from 43.8% to 55.6%, with logistic regression confirming statistically distinct trajectories between the 2 levels of care. Third, several years after the acute phase of the crisis, the proportion of negative reviews has not returned to prepandemic baseline, supporting the interpretation that the pandemic produced a persistent structural shift in citizen evaluation of the health system rather than a temporary disruption.

### Comparison With Prior Work

Prior to the pandemic, negative reviews accounted for approximately 38.7% of the corpus, a figure broadly consistent with patterns documented in studies of online health care reviews in comparable contexts [22]. Following the onset of the pandemic in early 2020, this proportion rose markedly to 73.7%, and although it declined slightly in subsequent years, it remained substantially elevated at 66.5% in the postpandemic period, well above prepandemic levels. The structural break detected around March 2020 using change-point analysis provides additional empirical support for the interpretation that the pandemic represented a discontinuity rather than an acceleration of preexisting trends.

Notably, the data also reveal earlier structural shifts in the prepandemic period, with change points identified around 2017 in primary care and around 2018 in hospital care. While the drivers of these earlier transitions fall outside the primary scope of this study, they suggest that deterioration in public perception of health services preceded the pandemic and that COVID-19 may have acted as an amplifier of tensions that were already emerging within the system [24].

### Temporary Shock vs Structural Transformation

The conceptual framework proposed in this study distinguishes between 2 broad trajectories following systemic shocks to health services. Under a temporary shock model, disruptions produce transient increases in dissatisfaction that gradually resolve as organizational pressures diminish and services recover [34]. Under a structural transformation model, the shock functions as a critical juncture that produces durable changes in how citizens interpret and evaluate their encounters with health institutions [35].

The empirical evidence aligns with the structural transformation scenario. Negative perceptions did not return to prepandemic levels following the acute phase of the crisis: the proportion of negative reviews remained at 66.5% in the postpandemic period, compared with 38.7% before the pandemic, a difference of nearly 28 percentage points. The change-point analysis corroborates this interpretation, identifying March 2020 as a structural break rather than a temporary perturbation in the time series [2]. The logistic regression models additionally indicate that the probability of a review being negative increased significantly over time, independently of the pandemic period categorization, suggesting that the deterioration in public perception cannot be attributed exclusively to the acute disruptions of 2020‐2022 but reflects a broader and more durable transformation in how citizens narrate their experiences of care.

This interpretation is consistent with broader theoretical perspectives on institutional legitimacy [36], which view large-scale crises as critical junctures capable of reshaping citizen expectations and trust in public institutions, particularly when disruptions affect the everyday interface between citizens and services. Once expectations are recalibrated downward and negative experiential frames become normalized, recovery to prior levels of perceived quality may require sustained organizational improvements rather than the mere resolution of the acute crisis [37].

At the same time, the observational design of the study does not permit strong causal attribution of the observed structural shift exclusively to the COVID-19 pandemic. Other concurrent factors, including ongoing reforms in primary care organization, sustained workforce shortages, the accelerated digitalization of public services, and broader societal changes in expectations regarding public institutions, may have contributed to the deterioration of citizen perception in parallel with the pandemic. The change-point analysis identifies March 2020 as a clear temporal discontinuity in the time series, strongly suggesting an association with the onset of the pandemic, but the persistence of elevated negative perception in subsequent years is likely to reflect a confluence of pandemic-related and structural drivers rather than a single causal mechanism.

### Divergent Trajectories Between Primary Care and Hospital Care

One of the most substantively important contributions of this analysis is the identification of divergent trajectories in public perception between primary care and hospital services. While previous studies examining patient experience during the COVID-19 pandemic have predominantly analyzed health systems as aggregate entities, the present findings indicate that the pandemic affected different levels of care in markedly distinct ways [2,38,39].

Hospital services experienced a moderate increase in negative perception during the pandemic (from 43.8% to 55.6%), and this remained relatively stable thereafter. Primary care, by contrast, underwent a substantially more pronounced and persistent deterioration (from 34.8% to 81.9%, remaining elevated at 75.7%). The interaction terms in the logistic regression models confirmed that these trajectories were statistically distinct.

This asymmetry suggests that the pandemic did not affect all components of the health system equally but rather amplified preexisting structural tensions at specific organizational interfaces. In the case of primary care, these tensions may be rooted in longstanding constraints, including insufficient staffing, growing demand associated with chronic disease management, and organizational fragmentation that rendered services particularly vulnerable to disruption under emergency conditions [40]. The earlier structural breaks identified in the change-point analysis, around 2017 in primary care and 2018 in hospital care, suggest that deterioration in public perception had already begun prior to the pandemic, consistent with documented pressures on primary care services in Spain during this period [20].

### Why Primary Care Absorbed the Negative Perception

From a structural perspective, primary care operates as the main interface between citizens and publicly funded health systems, characterized by frequent interactions, longitudinal relationships, and strong expectations of accessibility and continuity [41]. Unlike hospital care, typically encountered through episodic and acute episodes, primary care is embedded in the everyday routines of citizens, which exposes it disproportionately to reputational damage when accessibility is disrupted.

During the pandemic, primary care services in Spain underwent rapid and substantial organizational reconfiguration. The widespread adoption of telephone triage systems and remote consultation models, implemented almost overnight to manage patient flows and reduce transmission risk, fundamentally altered the experiential dimensions through which citizens interacted with primary care, particularly in terms of perceived accessibility, waiting times, and continuity of the doctor-patient relationship [42]. While these adaptations were organizationally necessary, they generated a significant mismatch between prior expectations and the experienced reality of care, which is likely to be reflected in the evaluative content of patient reviews [42].

A complementary explanation relates to the differential symbolic framing of care levels in public discourse during the pandemic. Hospitals were prominently positioned in media and institutional narratives as the front line of the crisis response, overloaded but indispensable [43]. Primary care, by contrast, was frequently experienced as less accessible and less visible [44], potentially functioning as a bottleneck through which broader systemic pressures were experienced and narrated by citizens, even when underlying constraints originated elsewhere in the system.

### Implications

These findings carry implications for understanding how health system legitimacy evolves following systemic shocks and for the design of postpandemic recovery strategies. While the present study does not directly measure care utilization or institutional trust, the sustained deterioration in public perception of primary care, the main everyday interface with publicly funded systems, may have downstream consequences for trust and engagement with health services that extend beyond the acute crisis period [41]. Persistent dissatisfaction with primary care access has been associated in the literature with behavioral responses, including bypassing the gatekeeping function, increasing reliance on emergency services, and reduced adherence to preventive care pathways [45], all of which carry significant implications for the efficiency and sustainability of publicly funded systems.

From a policy perspective, the persistence of elevated negative perception well into the postpandemic period suggests that recovery strategies should be explicitly designed to address the postcrisis phase rather than focusing exclusively on acute emergency response [46]. The deterioration in public perception did not resolve spontaneously as pandemic pressures diminished, implying that active and sustained intervention is required beyond the acute crisis. Recovery strategies focused exclusively on operational bottlenecks may fail to address the underlying erosion of experiential quality and relational trust that accumulated during the crisis [47,48], and rebuilding the communicative and relational dimensions of primary care, including continuity, personalized attention, and transparent communication with patients, may be as important as addressing structural capacity.

The divergence between primary and hospital care further points to the need for differentiated recovery strategies across levels of the system [49]. Investment should prioritize not only structural capacity, including staffing, infrastructure, and appointment systems, but also the relational dimensions of care that shape everyday citizen experience. The rapid adoption of remote consultation models during the pandemic, while organizationally necessary, may have produced lasting reputational costs that have not been fully reversed [42]. Policymakers should consider whether the current balance between remote and face-to-face consultation adequately meets citizen expectations and whether targeted efforts to restore in-person accessibility could contribute to rebuilding public trust. These considerations are particularly relevant in the Spanish context, where primary care has faced sustained pressure from workforce shortages, increasing demand, and organizational fragmentation predating the pandemic [20,40].

From a methodological standpoint, this study demonstrates the potential of large-scale digital trace data as a continuous and scalable instrument for monitoring public perception of health systems longitudinally. Unlike traditional patient satisfaction surveys, online reviews are generated continuously, accumulate across extended time periods, and capture unsolicited citizen discourse in naturalistic settings [6]. The approach developed here could be extended to other regional health systems or adapted to monitor specific domains of care quality over time. Future research could build on this work by incorporating fine-grained textual analysis of review content to identify the specific drivers of persistent dissatisfaction [2] and by conducting comparative analyses across health systems with different organizational models. Although the analysis presented here is grounded in the Andalusian context, comparable patterns of deteriorated patient experience during the pandemic have been documented in several other publicly funded health systems [22,23], suggesting that the dynamics described may reflect broader cross-system phenomena rather than purely local effects.

### Limitations

Several limitations should be considered when interpreting these findings. First, online reviews are subject to self-selection bias, as individuals who choose to leave reviews are more likely to report particularly positive or negative experiences. Users of online review platforms tend to be younger, more digitally engaged, and more urban, which may limit the representativeness of the sample with respect to the overall population of health service users, and prior research has documented a negativity bias in online reviews [50,51]. The analytical corpus therefore reflects the discursive expression of a self-selected subset of the population, and absolute levels of negativity should not be interpreted as population-representative estimates of citizen satisfaction. In addition, the volume of available reviews per facility was uneven across the analytical corpus, so that facilities with limited online presence or low review density contributed less information to the longitudinal comparisons, weighting the corpus toward facilities and time periods with higher review activity. The longitudinal design partly mitigates this concern by focusing on relative changes in proportions over time rather than absolute levels: under the assumption that the underlying biases in review behavior remained reasonably stable across the study period, temporal comparisons remain informative about shifts in public perception. The manual annotation used to validate the star-rating-based sentiment classification was performed by a single annotator, which precludes the assessment of interrater reliability; future validation work using multiple independent annotators would strengthen the methodological foundation of star-rating-based sentiment proxies in this type of corpus.

Second, the volume of online reviews increased substantially over the study period, which may reflect not only changes in user experience but also broader changes in platform adoption and user behavior, raising the possibility of time-varying measurement bias. To mitigate this concern, the analysis focused on the proportions of negative reviews rather than absolute counts, an approach that is robust to changes in overall review volume; in addition, the sharp discontinuity identified by the change-point analysis around March 2020 is difficult to attribute solely to gradual shifts in reviewer composition or in platform adoption, which would be expected to produce smooth secular trends rather than abrupt structural breaks. Nevertheless, it cannot be entirely ruled out that gradual changes in reviewer composition contributed in part to the observed trends. Within-year seasonality was also not explicitly modeled in the regression analyses, although the monthly aggregation used in the change-point detection partially captures sub-annual variation.

Third, the use of star ratings as a proxy for perception provides a unidimensional measure that does not capture specific domains such as communication, waiting times, or perceived quality of care. While the high agreement observed between star ratings and manually annotated sentiment supports their use as a scalable indicator, more granular analyses combining sentiment trajectories with aspect-based or thematic analysis of review content would be necessary to fully understand the specific experiential drivers of dissatisfaction.

Fourth, the statistical models explain only a limited proportion of the variability in individual-level outcomes, which is expected in analyses of subjective perceptions that are influenced by multiple contextual factors not captured in the dataset. The objective of the models was not to predict individual reviews but to estimate population-level effects and identify structural changes in perception over time.

Finally, the findings are drawn from a single regional health system, and their generalizability to other institutional or national contexts should be assessed through comparative research.

### Conclusions

Public perception of health care services did not recover to prepandemic levels in the years following the COVID-19 crisis. The evidence presented here indicates that the pandemic marked a structural turning point in how citizens evaluate publicly funded health services, with the structural break detected around March 2020 representing a clear discontinuity rather than a transient perturbation.

The findings reveal a marked divergence between primary care and hospital services that persisted well into the postpandemic period. While hospital services experienced a moderate and relatively stable increase in negative perception, primary care underwent a substantially more pronounced and enduring deterioration. This asymmetry suggests that the pandemic amplified preexisting structural tensions within primary care rather than affecting the health system homogeneously, and that the experiential and symbolic dimensions of primary care made it particularly vulnerable to reputational damage when everyday accessibility was disrupted.

These findings have direct implications for postpandemic recovery policy. The persistence of elevated negative perception years after the resolution of the acute crisis indicates that restoring service capacity alone is insufficient to rebuild public trust. Recovery strategies should explicitly target the relational, communicative, and accessibility dimensions of primary care and should be sustained beyond the acute phase of the crisis. Differentiated approaches across levels of care are necessary to avoid misallocation of resources and to address the specific deficits that have accumulated in primary care. Concrete examples of such differentiated recovery measures could include sustained investment in primary care workforce expansion to restore in-person accessibility, the introduction of explicit indicators of relational and communicative quality alongside operational efficiency metrics, and the development of mechanisms for periodic monitoring of citizen narratives as an early-warning instrument for shifts in perceived legitimacy.

Beyond the Andalusian context, these findings illustrate how systemic shocks to publicly funded services may produce durable shifts in citizen evaluation that outlast the operational resolution of the crisis itself, and they underline the need for health system governance frameworks to monitor and respond to the experiential and relational dimensions of care, not only the operational ones. The methodological framework developed here, combining large-scale digital trace data, validated sentiment classification, and change-point detection, is in principle transferable to any health system with sufficient digital review activity, offering a low-cost and continuous instrument that complements rather than replaces traditional satisfaction surveys and provides timely signals about shifts in citizen experience that would otherwise go undetected until the next formal measurement cycle. As health systems worldwide navigate postpandemic recovery, the capacity to detect early and persistent shifts in citizen discourse may become an increasingly relevant component of resilience and legitimacy monitoring in publicly funded health care.

## Supplementary material

10.2196/96824 Multimedia Appendix 1 Study flow diagram showing review retrieval, preprocessing, application of eligibility criteria, and construction of the final analytical corpus for the retrospective longitudinal observational study of public perception of the Andalusian Public Health System, Spain, 2016-2025.

10.2196/96824 Checklist 1 STROBE checklist.

## Abbreviations

BOJA: Official Gazette of the Regional Government of Andalusia
EU-GDPR: European General Data Protection Regulation
OR: odds ratio
STROBE: Strengthening the Reporting of Observational Studies in Epidemiology
