# Supplementary material for: Public Perception of Health Care Before, During, and After COVID-19: Longitudinal Analysis of Online Reviews
Source: J Med Internet Res. 2026 Aug 12;28:e96824. doi: 10.2196/96824 (PMC13465930; doi:10.2196/96824)
Supplement: Supplementary file 1 [file jmir-v28-e96824-s002.pdf]

STROBE Statement—checklist of items that should be included in reports of observational studies

	Item No	Recommendation	Reported on page / section
**Title and abstract**	1	(*a*) Indicate the study's design with a commonly used term in the title or the abstract	Title ("A Longitudinal Analysis…") and Abstract Methods ("A retrospective longitudinal observational study")
		(*b*) Provide in the abstract an informative and balanced summary of what was done and what was found	Structured Abstract with Background, Objective, Methods, Results, Conclusions.
**Introduction**			
Background/rationale	2	Explain the scientific background and rationale for the investigation being reported	Introduction (paragraphs on online reviews as data source, public perception and legitimacy, COVID-19 as systemic shock, and gap in literature).
Objectives	3	State specific objectives, including any prespecified hypotheses	Introduction, "Study objective and research question" subsection.
**Methods**			
Study design	4	Present key elements of study design early in the paper	Methods, "Study Design" subsection. Also includes STROBE adherence statement and reference to the completed checklist as Multimedia Appendix 1.
Setting	5	Describe the setting, locations, and relevant dates, including periods of recruitment, exposure, follow-up, and data collection	Methods, "Data Source and Data Collection" (Andalusia, Servicio Andaluz de Salud, publicly funded facilities) and "Web Scraping Procedure" (data extraction December 2025, analytical period 2016–2025).
Participants	6	(*a*) *Cohort study*—Give the eligibility criteria, and the sources and methods of selection of participants. Describe methods of follow-up<br>*Case-control study*—Give the eligibility criteria, and the sources and methods of case ascertainment and control selection. Give the rationale for the choice of cases and controls<br>*Cross-sectional study*—Give the eligibility criteria, and the sources and methods of selection of participants	Methods, "Data Source and Data Collection" (sampling frame: all publicly funded health care facilities in Andalusia from official institutional directory; no sampling applied) and "Text Preprocessing and data cleaning" (inclusion criteria: ≥5 words, Spanish language, text content present).
		(*b*) *Cohort study*—For matched studies, give matching criteria and number of exposed and unexposed	Not applicable; no matching procedure was performed.

		Case-control study—For matched studies, give matching criteria and the number of controls per case	
Variables	7	Clearly define all outcomes, exposures, predictors, potential confounders, and effect modifiers. Give diagnostic criteria, if applicable	Methods, "Sentiment Categorization" (outcome: review classified as negative when ≥4 stars vs ≤2 stars, with 3-star reviews treated as neutral) and "Statistical Analysis" (predictors: level of care, centered year, pandemic period; interactions specified). No formal confounders modelled given the descriptive longitudinal nature of the analysis.
Data sources/ measurement	8*	For each variable of interest, give sources of data and details of methods of assessment (measurement). Describe comparability of assessment methods if there is more than one group	Methods, "Web Scraping Procedure" (Selenium + Apify pipeline) and "Sentiment Categorization" (star ratings as sentiment proxy, validated against manual annotation of 300 reviews, Cohen's κ = 0.84).
Bias	9	Describe any efforts to address potential sources of bias	Methods, "Sentiment Categorization" (validation procedure addresses sentiment misclassification bias) and Discussion → "Limitations" subsection (self-selection bias, negativity bias, demographic skew of online reviewers, changes in platform adoption over time).
Study size	10	Explain how the study size was arrived at	Methods, "Data Source and Data Collection" subsection: justification of study size as a population-based census of all publicly funded facilities in Andalusia; no a priori sample size calculation was performed.
Quantitative variables	11	Explain how quantitative variables were handled in the analyses. If applicable, describe which groupings were chosen and why	Methods, "Statistical Analysis" (year handled as centered continuous variable; pandemic period as categorical with three levels; level of care as binary).
Statistical methods	12	(*a*) Describe all statistical methods, including those used to control for confounding	Methods, "Statistical Analysis" (logistic regression with progressively specified nested models).
		(*b*) Describe any methods used to examine subgroups and interactions	Methods, "Statistical Analysis" (interaction terms between level of care and year, and between level of care and pandemic period).
		(*c*) Explain how missing data were addressed	Methods, "Statistical Analysis" subsection (final paragraph): all analytical variables (star rating, date, facility identifier, level of care) were complete in the analytical corpus; reviews with missing or insufficient text were excluded during preprocessing; no imputation was required.
		(*d*) *Cohort study*—If applicable, explain how loss to follow-up was addressed	Methods, "Statistical Analysis" ("Robust standard errors clustered at the health care facility level were used for coefficient inference"), which accounts for non-independence of reviews within facilities.

		Case-control study—If applicable, explain how matching of cases and controls was addressed<br>*Cross-sectional study*—If applicable, describe analytical methods taking account of sampling strategy	
		(*e*) Describe any sensitivity analyses	Methods, "Statistical Analysis" subsection: progressive specification of nested logistic regression models and two complementary parameterizations of time (centered year and pandemic period) function as robustness checks for the stability of main associations across alternative analytical strategies.

**Results**			
Participants	13*	(a) Report numbers of individuals at each stage of study—eg numbers potentially eligible, examined for eligibility, confirmed eligible, included in the study, completing follow-up, and analysed	Methods, "Text Preprocessing and data cleaning" subsection (final paragraph). Flow diagram in Multimedia Appendix 2.
		(b) Give reasons for non-participation at each stage	Methods, "Text Preprocessing and data cleaning" subsection: reasons for exclusion explicitly listed at the preprocessing stage (absence of textual content, fewer than five words, non-Spanish language). Not further applicable: the study used unsolicited user-generated content and did not involve active recruitment of human participants, so traditional non-participation stages do not apply.
		(c) Consider use of a flow diagram	Flow diagram provided as Appendix 2
Descriptive data	14*	(a) Give characteristics of study participants (eg demographic, clinical, social) and information on exposures and potential confounders	Results, first paragraph (N=47,589 reviews from 812 facilities; 60.3% primary care, 39.7% hospitals; temporal distribution shown in Figure 1).
		(b) Indicate number of participants with missing data for each variable of interest	Not applicable; no missing data in the analytical corpus after preprocessing.
		(c) *Cohort study*—Summarise follow-up time (eg, average and total amount)	Methods, "Web Scraping Procedure" and Results (analytical period 2016–2025, ten years of continuous observation of all included facilities).
Outcome data	15*	*Cohort study*—Report numbers of outcome events or summary measures over time	Results, "Temporal evolution of negative perception" (overall proportions per period) and Table 1 (period × level of care breakdown).
		Case-control study—Report numbers in each exposure category, or summary measures of exposure	

		Cross-sectional study—Report numbers of outcome events or summary measures	
Main results	16	(*a*) Give unadjusted estimates and, if applicable, confounder-adjusted estimates and their precision (eg, 95% confidence interval). Make clear which confounders were adjusted for and why they were included	Results, "Logistic regression models" and Tables 2 and 3 (odds ratios with 95% CIs and p-values, robust standard errors clustered at facility level).
		(*b*) Report category boundaries when continuous variables were categorized	Methods, "Temporal Analysis" (pre-pandemic 2016–2019, pandemic 2020–2021, post-pandemic 2022–2025).
		(*c*) If relevant, consider translating estimates of relative risk into absolute risk for a meaningful time period	Not applicable; absolute proportions of negative reviews are reported directly alongside the relative measures (odds ratios) in Tables 1, 2, and 3.
Other analyses	17	Report other analyses done—eg analyses of subgroups and interactions, and sensitivity analyses	Methods, "Change-Point Detection" and Results, "Change-point detection" (PELT algorithm with RBF cost function as a structural-break analysis complementing the regression models).
**Discussion**			
Key results	18	Summarise key results with reference to study objectives	Discussion, "Principal Findings" subsection
Limitations	19	Discuss limitations of the study, taking into account sources of potential bias or imprecision. Discuss both direction and magnitude of any potential bias	Discussion, "Limitations" subsection (final subsection of Discussion after restructure), addressing self-selection bias, negativity bias, demographic skew, platform adoption changes, unidimensionality of star ratings, low pseudo-R^2, and single-region scope.
Interpretation	20	Give a cautious overall interpretation of results considering objectives, limitations, multiplicity of analyses, results from similar studies, and other relevant evidence	Discussion, "Comparison With Prior Work", "Temporary shock vs structural transformation", "Divergent trajectories between primary and hospital care", "Why primary care absorbed the negative perception", and "Implications" subsections.
Generalisability	21	Discuss the generalisability (external validity) of the study results	Discussion, "Limitations" (single-region scope) and "Implications" (transferability of methodological framework discussed in Conclusions).
**Other information**			
Funding	22	Give the source of funding and the role of the funders for the present study and, if applicable, for the original study on which the present article is based	Funding section at end of manuscript

*Give information separately for cases and controls in case-control studies and, if applicable, for exposed and unexposed groups in cohort and cross-sectional studies.

Note: An Explanation and Elaboration article discusses each checklist item and gives methodological background and published examples of transparent reporting. The STROBE checklist is best used in conjunction with this article (freely available on the Web sites of PLoS Medicine at http://www.plosmedicine.org/, Annals of Internal Medicine at http://www.annals.org/, and Epidemiology at http://www.epidem.com/). Information on the STROBE Initiative is available at www.strobe-statement.org.
